# Dose-volume histogram quality assurance for linac-based treatment planning systems

**DOI:** 10.4103/0971-6203.71759

**Published:** 2010

**Authors:** Michael S. Gossman, Morris I. Bank

**Affiliations:** Tri-State Regional Cancer Center, Medical Physics Section, 706, 23^rd^ Street, Ashland, Kentucky, USA; 1 St. Vincent Oncology Center, 8301 Harcourt Road, Indianapolis, Indiana, USA

**Keywords:** Dose, dose–volume, dose–volume histogram, histogram, quality assurance, treatment planning system, Volume

## Abstract

Dose–volume histograms provide key information to radiation oncologists when they assess the adequacy of a patient treatment plan in radiation therapy. It is important therefore that all clinically relevant data be accurate. In this article we present the first quality assurance routine involving a direct comparison of planning system results with the results obtained from independent hand calculations. Given a known three-dimensional (3-D) structure such as a parallelepiped, a simple beam arrangement, and known physics beam data, a time-efficient and reproducible method for verifying the accuracy of volumetric statistics (DVH) from a radiation therapy treatment planning system (TPS) can be employed rapidly, satisfying the QA requirements for (TPS) commissioning, upgrades, and annual checks. Using this method, the maximum disagreement was only 1.7% for 6 MV and 1.3% for 18 MV photon energies. The average accuracy was within 0.6% for 6 MV and 0.4% for 18 MV for all depth-dose results. A 2% disagreement was observed with the treatment planning system DVH from defined volume comparison to the known structure dimensions.

## Introduction

A qualitative isodose distribution superpositioned over computed tomographic (CT) data is often insufficient to provide qualitative data for the Radiation Oncologist to determine the adequacy of a patient treatment plan. A more quantitative result is necessary which modern treatment planning systems provide in statistical analysis plots known as dose–volume histograms (DVHs). These plots describe the dose throughout the volume of each structure contoured within the plan. Since it is important to have accurate statistics for clinical use, it is important to test the system against an independent source. Concise summary data verifications for the 3-D dose distributions are needed.[1] A literature search shows a lack of any quality control procedures performed and described to ensure that computerized DVH analyses are correct.[2,3] Our research provides this methodology and discussion on the accuracy of its use.

Often the statistics of the dose-to-volume relationship are the deciding factor when one plan is chosen over another.[4] However, this is not always true since the DVH has no spatial resolution.[5] The criterion of dose–volume constraints may also be associated with the initial prescription. In order to verify dose–volume (DVH) statistics from a treatment planning system, a known volume must be used. A complete understanding of the mathematical components involved, the ‘untimely’ cost of analysis, and the methodic conceptual construction of a valid quality assurance test has kept such research from being published.[1] Quality assurance of the DVH has been strongly recommended though the authors do not explain exactly how this is to be done.[3,6–8] This article presents a reproducible and efficient DVH quality assurance process, with a clear step-by-step description of the methodology.

Using a commercial radiation therapy treatment planning system and given an artificial CT data set representing a homogeneous water phantom, one may construct a single treatment plan that can be utilized annually or when the TPS is updated. In the method discussed in this article the majority of effort is involved with the initial contouring of a structure with a known volume and the associated single-field setup. Here, we chose a rectangular parallelepiped or ‘block.’ The results of volumetric dose are mathematically calculated from depth-dose equations and then directly compared to the results from the treatment planning system DVH plots. Being spreadsheet based, this methodology is repeatable when needed, merely requiring a recalculation of the computer treatment plan. From each new calculation, DVH data can be extracted and input for direct comparison. The method described complements the focus of attention on DVH errors, by avoiding the effects due to grid size and sampling frequency problems. Further, it provides volumetric and geometric resolution, compares absolute and relative dose results, and tests statistics generated for dose maxima, minima, and partial volume doses within the contoured structure.

## Materials and Methods

### Object and beam definitions

The Eclipse Treatment Planning System (Varian Medical Systems, Inc.) was used to define the volumes of interest and for dose delivery simulation. A homogeneous phantom set to density of 1.0 resembling tissue equivalent media was chosen for use with all treatment planning experiments. The phantom consisted of an artificial data set created within the treatment planning software. The dimensions of the phantom data set were suitable for radiation fields of all sizes at 40 × 40 cm^2^. A slice spacing of 1.25 mm was chosen for data usage.

The Eclipse software was commissioned to compute modeled dose from a 21EX Clinac (Varian Medical Systems, Inc.) using the Anisotropic Analytical Algorithm (AAA) version 8.6 in External Beam Planning Software build 8.6.17. Since the 21EX particle accelerator was engineered to generate photon beams at 6 MV and 18 MV, both energy modalities were used. Each beam was calibrated in accordance with the clinical reference dosimetry standard: the American Association of Physicists in Medicine (AAPM) TG-51 formalism.[9] For all photon beams at this facility, a calibration was performed such that 1.00 cGy/MU was achieved at the source-axis distance (SAD) of the LINAC and at the depth where the maximum dose was achieved.

Using the contouring workspace in the software, an object contour was created on one superior slice of the CT data of the water phantom. The contour was given dimensions of 2 cm × 10 cm. The square object drawing was copied and reapplied to slices consecutively inferior in the phantom material through an additional 2 cm. A total of 17 slices were contoured using the ‘copy and paste’ tool, since (1 slice/0.125 cm) × (2 cm – 0 cm) + 1 = 17 slices. The resulting structure is a rectangular solid known as a parallelepiped, with dimensions of 2 × 2 × 10 cm^3^. This 40 cm^3^ known structural volume was used for dose analysis. The phantom was assigned a Hounsfield unit (HU) value of 0, making it a unit density homogeneous phantom material.

After completing the contouring required, a treatment field was assigned to the CT data set. The isocenter of the field was centered at a depth of 10 cm in the water phantom (5 cm deep in the parallelepiped). The calculation reference point was at the same point. The prescribed dose to that point was 100 cGy. The field size of the beam was 30 × 30 cm^2^. For Varian standard scaling, the gantry angle was 180°, with the collimator angle and the couch angle each set at 180°. The field was copied so that both 6 MV and 18 MV results could be tested independently. The volume calculation grid at 0.15 cm was chosen to be small, since large voxel sizes result in less accurate results.[10] Once all parameters in the plan were correctly verified, the dose throughout the 3-D volume was calculated..

### Hand calculations

**Figure 1:**
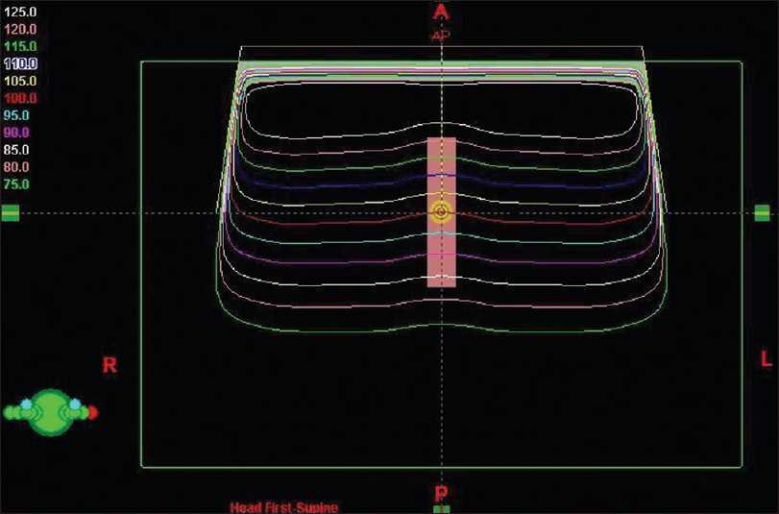
Axial view isodose distribution indicating the position of the rectangular volume within the water phantom. The isodose levels represent the percentage of dose, relative to 100 cGy at 10 cm phantom depth (5 cm structure depth), for an 18 MV beam having normal geometry and a field size of 30 × 30 cm^2^ at the SAD.

**Figure 2:**
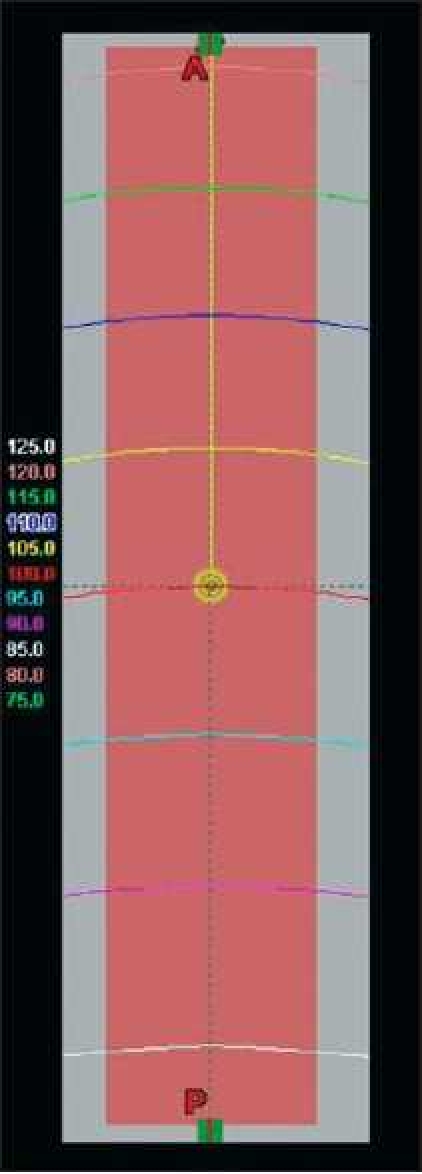
As in the previous illustration [Figure 1], this is a zoom-in on the distribution of dose that is seen inside the structure in the axial view

The resulting computation of dose is qualitatively exhibited in Figure 1. It is again shown in the following illustration with a focus on the region comprising the contoured structural volume [Figure 2]. Isodose lines ranging from 75% to 125% (in 5% increments) are displayed in the Figures 1 and 2 as seen from the planning software for 18 MV photons. The depth of dose occurrence is analyzed for accuracy against the known dose data commissioned for the planning system. The accuracy of the analysis is strictly a function of knowing the depth-dose for that point. Further knowledge of the precise volume of the contour receiving such dose at each depth permits taking into account field edge horn effects created by the flattening filter of the accelerator. The calculation of the number of monitor units necessary to arrive at this prescription dose is shown in Equation 1.

(1) $$\mathrm{MU}\;≅\;\frac{\mathrm{TD}}{\mathrm{Output}\times {\mathrm{TMR}}_{d,{\mathrm{FS}}_{d}}\;\times {\mathrm{Sc}}_{{\mathrm{FS}}_{\mathrm{col}}}\;\times {\mathrm{Sp}}_{{\mathrm{FS}}_{d}}}$$

The number of monitor units (*MU*) is determined from the quotient of the prescribed tumor dose (*TD*) with the product of four variables: the machines calibration dose-rate (*Output*), the tumor maximum dose ratio (*TMR_d, FSd_*), the collimator head scatter factor (*Sc_FScol_*), and the phantom scatter factor (*Sp_FSd_*). For this exercise, the tumor is the contoured block. The calculation depth is denoted *d*, with *FS_col_* representing the collimator field size and *FS_d_* representing the field size at that depth. For the depth of 10 cm in the water phantom (5 cm within the block contour) the dose to the point of interest is 100 cGy. For all other depths (*d’*), the percentage depth-dose (*%DD*) along the central ray may be calculated from Equation 2:

(2) $$\%\mathrm{DD}\;≅\;100\;\times \;\frac{\mathrm{MU}\times \mathrm{Output}\times {\mathrm{TMR}}_{d\prime ,{\mathrm{FS}}_{d\prime }}\;\times {\mathrm{Sc}}_{{\mathrm{FS}}_{\mathrm{col}}}\;\times {\mathrm{Sp}}_{{\mathrm{FS}}_{d\prime }}\;\times {\left(\frac{\mathrm{SAD}}{\mathrm{SSD}\;+\;d\prime }\right)}^{2}}{\mathrm{TD}}$$

The source–axis distance (*SAD*) defined as the distance from the source of radiation to the center of gantry rotation on the accelerator is precisely 100 cm. The source–surface distance (*SSD*) is the distance from the source to the phantom surface. The distance was fixed in this study at 90 cm.

It is expected that the 100% isodose line will pass through the centroid of the contoured parallelepiped. As shown in Figure 2, the horn effects of the planning field clearly present a distribution of dose that is curved, such that the amount of volume receiving 100% of the dose is more than half of the object volume. Although the field generally exhibits flatness and symmetry within industry standards, this is not to be confused with the intent of analytically accounting for the change in volume receiving the dose inside of the isodose line associated at that depth. Off-axis ratios (*OAR*) from scanned data were used to more accurately quantify the contour volume receiving the dose at each depth reviewed. These were used to correct the geometric volume of the object for accuracy. From the dimensions of the contour described, it was possible to study the accuracy of dose mathematically from scanned data *vs* the treatment planning system’s DVH results. Dose depth studies here range from a phantom depth of 6 cm (1 cm within the block contour) to a phantom depth of 13 cm (8 cm within the contour).

### Computer calculations

The statistical DVH computed by the treatment planning software was created through a lengthy interpolation process. First, the shape of the parallelepiped was registered in 3-D coordinates space along with the dose values for each voxel. By binning each voxel, a statistical account of the dose received to each segment of the structure can be determined by weighting the dose of each bin with neighboring bins throughout the entire object space. This iterative weighting is referred to as the dose matrix coordinates. It is within the dose matrix that differences in the binned object volume calculation are observed as a source of error. Imported data from CT acquisition is a common cause of re-sampling errors, where the dose matrix and the dose grid chosen for computation are considerably different. For quality assurance studies such as this, we recommend the creation of an artificial data set to avoid the significant levels of error that could be otherwise introduced. Finally, the cumulative DVH is generated as an integral of the sampled dose over the interpolated structures.[11]

## Results

Using a spreadsheet for calculations, all of the parameters discussed here were imbedded. The dose was determined at each depth using Equations 1 and 2. Since 100% of the dose is normalized to a single point in the center of the block, half of it can be expected to receive the full prescribed dose along with a subtle increase due to horn effects that is accounted for using OARs. Thus, the dose was renormalized at each depth in accordance to the prescription. At 10 cm in the phantom (5 cm in the block contour) the dose should be 100 cGy. All other depth-doses are relative to the dose at this point. Therefore, each percentage depth-dose was renormalized with respect to the weight point. Points anterior to this resulted in doses greater than 100 cGy, whereas points downstream resulted in doses less than 100 cGy.

The fractional volume known was mathematically determined at each depth. Dose to each tenth fraction of volume was calculated from knowledge of the isodose line traversed at each depth. Then, while taking this result in product with a percentage increase in the dose attributed to the horn effect within the beam profile, the resulting percentage of volume enveloping the isodose line at each depth is evident.

**Figure 3:**
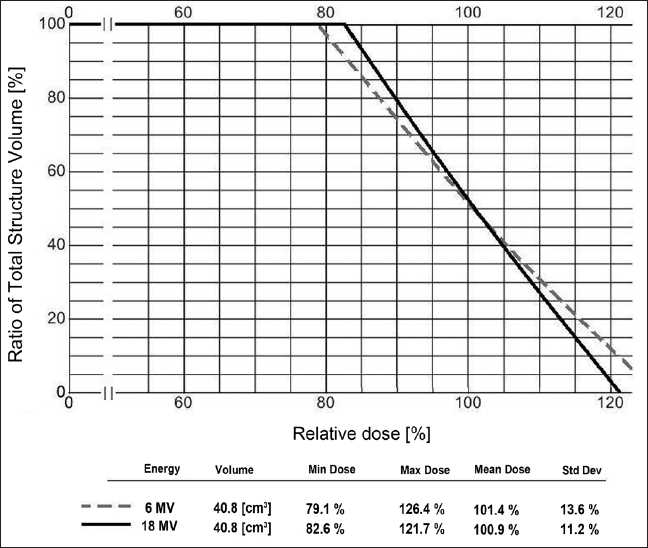
Dose-volume histogram statistical analysis from the treatment planning system for 6 MV (dashed) and 18 MV (solid)

The percentage of volume encompassing the dose at each depth as well as the stated renormalized percentage depth-dose at each volumetric depth was calculated and directly compared to the results from the planning system DVH plots. Both X-ray energy modalities were reviewed independently and identically in the planning system. The treatment planning system DVH statistics for 6 MV and 18 MV are presented in Figure 3. A sample of the resulting calculation routine screen is presented in Table 1.

**Table 1:** Spreadsheet of independent dose–volume calculation correlation to treatment planning system DVH results. The independent calculations appear on the left of the spreadsheet, with DVH results on the right along with their overall agreement. Both 6 MV and 18 MV results are presented.

*6MV*	*d (cm)*	*FS*	*FS (d)*	*TMR (d, FSd)*	*<OAR>*	*Sc (FS)*	*Sp (FSd)*	*Ií2*	*MU*	*Dose (cGy)*	*% Vol (at d) Shape*	*% Vol (for D) c OAR*	*% D (at d) Calc*	*% D (at d) DVH*	*% V (at d) DVH*	*D/V (Calc/DVH) %*
6 MV																
	6	30.0	28.8	0.917	1.001	1.047	1.025	1.085	112.2	119.8	10	10.01	119.8	120.5	9.9	-1.7
	7	30.0	29.1	0.895	1.001	1.047	1.025	1.063	112.2	114.5	20	20.02	114.5	115.1	20.0	-0.8
	8	30.0	29.4	0.873	1.001	1.047	1.025	1.041	112.2	109.4	30	30.04	109.4	110.1	29.9	-0.9
	9	30.0	29.7	0.852	1.001	1.047	1.026	1.020	112.2	104.7	40	40.05	104.7	105.0	39.9	-0.6
	10	30.0	30.0	0.830	1.001	1.047	1.026	1.000	112.2	100.0	50	50.06	100.0	100.2	50.0	-0.4
	11	30.0	30.3	0.807	1.001	1.047	1.026	0.980	112.2	95.3	60	60.07	95.3	95.7	60.0	-0.5
	12	30.0	30.6	0.785	1.001	1.047	1.026	0.961	112.2	90.9	70	70.08	90.9	91.1	70.0	-0.4
	13	30.0	30.9	0.763	1.001	1.047	1.026	0.943	112.2	86.7	80	80.10	86.7	86.9	80.0	-0.4
	14	30.0	31.2	0.741	1.001	1.047	1.026	0.925	112.2	82.5	90	90.11	82.5	82.8	90.0	-0.5
	15	30.0	31.5	0.721	1.001	1.047	1.027	0.907	112.2	78.9	100	100.12	78.9	78.8	100.0	-0.0
18 MV															
	6	30.0	28.8	0.965	1.000	1.080	1.008	1.085	102.2	116.3	10	10.00	116.3	117.1	9.9	-1.3
	7	30.0	29.1	0.949	1.000	1.080	1.008	1.063	102.2	112.0	20	20.00	112.0	112.6	20.1	-0.2
	8	30.0	29.4	0.933	1.000	1.080	1.008	1.041	102.2	107.9	30	30.00	107.9	108.3	30.0	-0.4
	9	30.0	29.7	0.918	1.000	1.080	1.009	1.020	102.2	104.1	40	40.00	104.1	104.2	40.0	-0.2
	10	30.0	30.0	0.898	1.000	1.080	1.009	1.000	102.2	100.0	50	50.00	100.0	100.3	50.1	-0.2
	11	30.0	30.3	0.882	1.000	1.080	1.009	0.980	102.2	96.1	60	60.00	96.1	96.6	60.0	-0.4
	12	30.0	30.6	0.866	1.000	1.080	1.010	0.961	102.2	92.6	70	70.00	92.6	93.3	69.9	-0.5
	13	30.0	30.9	0.849	1.000	1.080	1.010	0.943	102.2	89.0	80	80.00	89.0	89.5	80.0	-0.5
	14	30.0	31.2	0.833	1.000	1.080	1.010	0.925	102.2	85.7	90	90.00	85.7	86.1	90.0	-0.5
	15	30.0	31.5	0.818	1.000	1.080	1.011	0.907	102.2	82.6	100	100.00	82.6	82.7	99.9	-0.2

Data presented in the table on the left are utilized in the calculation of dose at the prescription depth point of interest, which is shown in the middle of the spreadsheet. From Equation 2, the ratio of the calculated point dose to the 100 cGy prescribed dose is indicated as *%DD_d_^calc^*. The percentage of geometric volume receiving that dose is given as %V*_d_^Calc^*. The object volume and beam assignment were chosen such that the increase in volume is in the anterior to posterior direction. This is the same directional path of the radiation beam. Therefore, %V*_d_^Calc^* is determined knowing the geometric parallelepiped volume encompassed at each depth along with a residual amount to account for the curvature of the isodose line. The additional volume correction can be determined by averaging the OARs from the central axis through the half-width of the object diameter. The object is only 5 cm wide, so off-axis factors are only considered up to 2.5 cm. At 6 MV and 18 MV, the average OAR was determined to be 1.001 and 1.000, respectively.

Data analyzed from the treatment planning software are revealed in Table 1 on the right. The DVH computed percentage depth-dose at the calculation point is denoted %*DD*_d_^DVH^. The percentage of reconstructed volume receiving that dose is given as %V*_d_^DVH^*. The calculated percentage depth-doses and the software computed percentage depth-doses were compared relative to the volume encompassed at each depth in the object. The results yield a maximum deviation $\left(\%\delta_{\mathrm{DVH}}^{\mathrm{Calc}}\right)$ of –1.7% for 6 MV and –1.3% for 18 MV. Equation 3 below indicates the calculation performed.

(3) $$\%\delta_{\mathrm{DVH}}^{\mathrm{Calc}}\;≅\;\frac{\left[\frac{\%{\mathrm{DD}}_{d\prime }^{\mathrm{Calc}}}{\%V_{d\prime }^{\mathrm{Calc}}}\right]}{\left[\frac{\%{\mathrm{DD}}_{d\prime }^{\mathrm{DVH}}}{\%V_{d\prime }^{\mathrm{DVH}}}\right]}\times 100-100$$

As compared to the manually created known volume of 40.0 cm^3^, the DVH indicated a total volume of 40.8 cm^3^. This is within tolerance at 2.0%. All data statistics were seen to satisfy requirements of analysis for the treatment planning software utilized here. In general, if independent calculations differ by more than 5.0%, the disparity should be investigated further and resolved.[12]

## Discussion and Conclusions

For quality assurance in medical physics practice, it is often advantageous to employ reproducible routines for calculations. Such is the case for software treatment planning system annual quality assurance testing. Here, it is shown how a routine can be created to test the treatment planning system on its ability to accurately calculate dose to a manually created parallelepiped volume and properly quantify the volumetric statistics from its computations. Dose-volume histograms are readily available in most clinically available TPS software. As important as statistical data are to radiation oncologists in this era, it is beneficial to understand the accuracy of such quantitative results upon which clinical decisions are based. The methods described here are reproducible, accurate, and serves as an effective tool to perform periodic quality assurance of the treatment planning system. Depending on the accuracy of the scanning data employed into the treatment planning system and the algorithms to which the DVHs are computed, the accuracy of the system could ideally be verified to within a few percent.

The planning DVH ties together the percentage of total structure volume to the percentage of relative dose it receives. The ratio of the two can be directly compared to the results from independent calculations. Here, a maximum deviation of 1.7% was observed and accepted for both 6 MV and 18 MV X-rays in this quantitative study. The accuracy average difference was 0.6% for 6 MV and 0.4% for 18 MV. The total volume difference for the 40 cm^3^ block structure was 2%.

This research describes a method which is ideal for validating the accuracy of the DVH statistical analysis in a commercially available radiation therapy treatment planning system. It is not necessary to recreate the entirety of the plan for the next test scheduled. It is only necessary to recalculate the dose using the system algorithm of choice. Further, the implementation of a spreadsheet to expedite the presented calculations can permit complete dose–volume histogram quality assurance in a timely manner. This research provides a sound basis for performing quality assurance tests for treatment planning system dose–volume histograms with successful results. Such quality assurance should be performed at the time of commissioning, annually, and after any major algorithm upgrades.
